# Association Between the Concentration and Rangeability of Cystatin C and Mortality of COVID-19 Patients With or Without Type 2 Diabetes Mellitus: A Retrospective Analysis

**DOI:** 10.3389/fendo.2021.642452

**Published:** 2021-06-21

**Authors:** Lei Yang, Dou Xu, Yiqing Tan, Bolin Li, Dan Zhu, Jingbo Wang, Hui Sun, Xinglong Liu, Xiaopu Zheng, Ling Zhu, Zhongyu Li

**Affiliations:** ^1^ Department of Cardiology, The First Affiliated Hospital of Xi’an Jiaotong University, Xi’an, China; ^2^ School of Software Engineering, Xi’an Jiaotong University, Xi’an, China; ^3^ Department of Radiology, Wuhan Third Hospital, Tongren Hospital of Wuhan University, Wuhan, China; ^4^ Department of Radiology, Shanghai Ninth People‘s Hospital Affiliated to JiaoTong University School of Medicine, Shanghai, China; ^5^ SenseTime Research, Beijing, China

**Keywords:** COVID-19, cystatin C, renal function, all-cause death, type 2 diabetes mellitus

## Abstract

**Background:**

We investigated if the concentration and “rangeability” of cystatin C (CysC) influenced the prognosis of coronavirus disease 2019 (COVID-19) in patients suffering from, or not suffering from, type 2 diabetes mellitus (T2DM).

**Methods:**

A total of 675 T2DM patients and 572 non-T2DM patients were divided into “low” and “high” CysC groups and low and high CysC-rangeability groups according to serum CysC level and range of change of CysC level, respectively. Demographic characteristics, clinical data, and laboratory results of the four groups were analyzed.

**Results:**

COVID-19 patients with a high level and rangeability of CysC had more organ damage and a higher risk of death compared with those with a low level or low rangeability of CysC. Patients with a higher level and rangeability of CysC had more blood lymphocytes and higher levels of C-reactive protein, alanine aminotransferase, and aspartate aminotransferase. After adjustment for possible confounders, multivariate analysis revealed that CysC >0.93 mg/dL was significantly associated with the risk of heart failure (OR = 2.231, 95% CI: 1.125–5.312) and all-cause death (2.694, 1.161–6.252). CysC rangeability >0 was significantly associated with all-cause death (OR = 4.217, 95% CI: 1.953–9.106). These associations were stronger in patients suffering from T2DM than in those not suffering from T2DM.

**Conclusions:**

The level and rangeability of CysC may influence the prognosis of COVID-19. Special care and appropriate intervention should be undertaken in COVID-19 patients with an increased CysC level during hospitalization and follow-up, especially for those with T2DM.

## Introduction

In late December 2019 in Wuhan City (China), many patients with undefined pneumonia were reported to be infected with a novel coronavirus. The latter was named “severe acute respiratory syndrome coronavirus 2” (SARS-CoV-2) (1–3). The disease that SARS-CoV-2 causes was named “coronavirus disease 2019” (COVID-19). The pandemic of COVID-19 has now infected over 170 million people worldwide until June of 2021 (4).

Kidney involvement is frequent in COVID-19; >40% of cases have abnormal proteinuria at hospital admission (5). Acute kidney injury (AKI) has been observed in 3%–15% of COVID-19 patients (6) and ≤25% in critically-ill COVID-19 patients (7). Greater possibility can be presented for AKI among patients with severe acute respiratory distress syndrome (ARDS), which necessitates invasive mechanical ventilation, especially for older patients or those with comorbidities such as hypertension or type 2 diabetes mellitus (T2DM) (8). Recent studies have suggested that acute renal damage can occur in the early stages of COVID-19, which is associated with death (5, 9, 10).

Xu G and colleagues stated that preexisting renal disease can be associated with an increased risk of adverse outcomes (adjusted hazard ratio of 2.0 (95% confidence interval (CI), 1.32–3.15)) for hospitalized patients (5). That study emphasized that AKI and severe kidney disease are independent risk factors for a poor prognosis of COVID-19, but included indices representing deterioration of renal function - rapid development of urea nitrogen and creatinine in this study. However accumulating evidence has shown that these biomarkers are suboptimal to detect kidney disease in early stages (11).

In the past decade, various studies have been proposed in measuring the serum level of certain biomarkers for early diagnosis of AKI (12), among which major attention has been drawn to cystatin C (CysC) (13). CysC is an early diagnostic biomarkers of AKI in different settings. Evidence points out that it is present approximately 2 days before the clinical syndrome of AKI develops (14), which has been shown to be useful in diagnosing AKI and predicting its outcomes (15).

Whereas the contribution of CysC and changes in the level of CysC with respect to outcomes in COVID-19 patients are poorly understood.

Like many countries, China has an aging population, and an increasing population of T2DM patients. The latter are more likely to develop COVID-19 and have worse clinical outcomes in comparison with COVID-19 patients not suffering from T2DM (16). We hypothesized that CysC and changes in the levels of CysC are associated with worse organ function and a higher risk of death in COVID-19 patients.

We focused on COVID-19 patients with T2DM and investigated the association between CysC and changes in CysC levels with regard to clinical outcomes. Moreover, we investigated whether the relationship between CysC and changes in CysC levels and COVID-19 prognosis differed between T2DM and non-T2DM patients.

## Methods

### Ethical Approval of the Study Protocol

This was a retrospective study conducted in Wuhan Third Hospital (Wuhan, China). The study protocol was approved by the Ethics Committees of Wuhan Third Hospital.

### Study Population and Data Collection

We considered all consecutive patients with severe COVID-19 admitted to Wuhan Third Hospital between January 11 to March 1, 2020. All patients were diagnosed based on the recommendations (fifth edition) set by the National Institute for Viral Disease Control and Prevention in China. Patients were included in this study if they met the following requirements: (1) 18 years of age or older; (2) had a contact history with patients diagnosed with COVID-19 and presented with the typical features of COVID-19 on computed tomography (CT); (3) a positive result for the nucleic acids of SARS-CoV-2 on the polymerase chain reaction test.

Exclusive criteria of this study: (1) missing data on renal chemistries (creatinine, urea nitrogen, CysC), important clinical covariates such as inflammation indicator, liver function, and hospital treatment; (2) had end-stage kidney disease (ESKD), (3) had prior kidney transplant; (4) had <2 serum CysC levels during admission; (5) with elevated glucose (fasting blood glucose ≥6.1 mmol/L or random glucose ≥11.1 mmol/L) from the nondiabetic group; and (6) type 1 diabetes. A total of COVID-19 patients were included in the current study.

Demographic characteristics, clinical information, laboratory test results, and clinical-outcome data were obtained using data collection forms from electronic medical records. The duration of disease onset to hospital admission and hospital discharge or death was recorded. Information recorded included demographic data, medical history, underlying comorbidities, laboratory findings (e.g., random blood glucose on admission, CysC level duration of hospital stay), and drugs (e.g., insulin and antihyperglycemic agents). Fasting plasma glucose (FPG) was measured for all patients during hospitalization. Comorbidities, including diabetes, cerebral diseases, cardiovascular diseases, and chronic renal diseases were defined as documented history in the admission notes. Cerebral diseases refer to cerebral infarction, epilepsy, Alzheimer’s disease, and Parkinson’s disease. Cardiovascular diseases refer to hypertension, coronary heart disease, arrhythmia, cardiomyopathy, and heart failure. Chronic renal diseases refer to chronic renal insufficiency, chronic renal failure, chronic nephritis, and nephrotic syndrome.

### Definitions

We encountered many difficulties in justifying requests for some tests during the outbreak of COVID-19. Oral glucose tolerance tests to diagnose T2DM and glycosylated hemoglobin were not routinely requested since they were considered low priorities for COVID-19 patients, making the diagnosis of new onset T2DM impossible. We therefore included patients with known T2DM history in the diabetes group and excluded patients with elevated glucose (fasting blood glucose ≥6.1 mmol/L or random glucose ≥11.1 mmol/L) from the nondiabetic group to make the analysis more coherent. The severity of kidney injury was denoted by the CysC level, which was measured upon hospital admission. Changes in renal function were defined as “CysC rangeability”, which was calculated as the difference in the CysC level between the time of hospital admission and the highest CysC level recorded during hospitalization. “Severe inflammation” was defined as the highest neutrophil:lymphocyte ratio >6.11 during hospitalization (17). “Liver injury” was defined as degree of alanine transaminase (ALT) ≥the upper limit of normal. ALT was selected to represent liver injury rather than aspartate transaminase (AST) due to the more predominant extra-hepatic sources of AST rendering it less liver-specific (18). “Heart failure” was defined as clinical symptoms (e.g., breathlessness, ankle swelling, and fatigue) are present with the level of N terminal pro B type natriuretic peptide (NT-proBNP) >300 pg/mL during hospitalization (19).

### Statistical Analyses

Continuous variables are presented as the mean ± SD if they have a normal distribution or median (lower quartile, upper quartile) value if they do not have a normal distribution. The Shapiro–Wilk test was used to test for normality. Categorical variables are presented as numbers and percentages. Differences in parameters among groups were analyzed using the Student’s *t*-test for variables with a normal distribution. The Mann–Whitney *U*-test was employed for continuous variables with a non-normal distribution, and the Chi-square test was used for categorical variables. Logistic regression models were used in univariate analyses and multivariate analyses to determine the prognostic value of the CysC level. Multivariate analyses were adjusted for significant baseline variables and the factors closely related to the outcome of patients with cardiovascular disease (e.g., age, sex, severe pneumonia, serum level of albumin, blood glucose, and log-BNP).

## Results

### Characteristics of Hospitalized Patients With COVID-19

A total of 1247 COVID-19-associated hospitalized adults from Wuhan City formed the study cohort. The median age was 63 (interquartile range, 51–70) years. Also, 9.4% of the COVID-19 patients were diagnosed with severe pneumonia and 48% were men. There were 675 patients with T2DM and 572 patients did not have T2DM. Severe pneumonia was diagnosed in 8.6% of non-T2DM patients and 10.2% of T2DM patients (**Table 1**).

**Table 1:** Characteristics of hospitalized patients with COVID-19.

Variables	Total	Non-T2DM	T2DM	p
	(n = 1247)	(n = 572)	(n = 675)	
Age	63 (51, 70)	66 (55, 72)	61 (47.5, 68)	<0.001
Men	598 (48)	270 (47)	328 (49)	0.53
Severe pneumonia, n (%)	118 (9.4)	49 (8.6)	69 (10.2)	0.285
CysC baseline, mg/L	0.93 (0.77, 1.14)	0.96 (0.79, 1.19)	0.9 (0.74, 1.12)	<0.001
Hospital stays (days)	15 (11, 17)	16 (14, 17)	14 (11, 17)	0.003
Glucocorticoid use				0.362
0 (no)	1123 (90%)	520 (91.0)	603 (89.3)	
1 (yes)	124 (10.0)	52 (9.0)	72 (10.7)	
Comorbidities				
Cerebral diseases, n (%)				0.003
0 (no)	1193 (95.7)	561 (98)	632 (93.6)	
1(yes)	54 (4.3)	11 (2.0)	43 (6.4)	
Cardiovascular diseases, n (%)				0.045
0 (no)	761 (61.0)	373 (65.2)	388 (57.5)	
1 (yes)	486 (39.0)	199 (34.8)	287 (42.5)	
Chronic renal diseases, n (%)				0.576
0 (no)	1221 (97.9)	565 (98.8)	656 (97.2)	
1 (yes)	26 (2.1)	7 (1.2)	19 (2.8)	
Complications during hospitalization				
Liver injury, n (%)				0.02
0 (no)	989 (79)	486 (85)	503 (75)	
1 (yes)	258 (21)	86 (15)	172 (25)	
Heart failure, n (%)				0.318
0 (no)	1203 (96)	548 (96)	655 (97)	
1 (yes)	44 (4)	24 (4)	20 (3)	
Severe inflammatory, n (%)				<0.001
0 (no)	1057 (85)	516 (90)	541 (80)	
1 (yes)	190 (15)	56 (10)	134 (20)	
All-cause death, n (%)				<0.001
0 (no)	1191 (96)	572 (100)	619 (92)	
1(yes)	56 (4)	0 (0)	56 (8)	

Data are reported as mean ± SD, median (IQR) or number and percentage. T2DM, type 2 diabetes mellitus; NT-proBNP, N terminal pro B type natriuretic peptide; CysC, cystatin C; BNP, brain natriuretic peptide. Severe inflammation was defined as the highest neutrophil:lymphocyte ratio >6.11 during hospitalization; liver injury was defined as a level of alanine transaminase >40 U/L at any time during hospitalization; and heart failure was defined as clinical symptoms present with the level of NT-proBNP >300 pg/mL during hospitalization.

The entire cohort was divided into two groups according to T2DM or the absence of T2DM. Laboratory testing comprised routine hematology, organ function (heart, liver, kidney), coagulation function, and infection indicators (**Table S1**). Compared with the non-T2DM patients, patients with diabetes were older and were more likely to have cerebral diseases and cardiovascular disease, and the prevalence of severe inflammation and liver injury in hospital was higher in T2DM patients. Neither severe pneumonia, heart failure in hospital, nor other comorbidities were significantly different between patients with T2DM and those without. Patients with T2DM also had a shorter duration of hospital stay (DoHS). T2DM contributed to the prevalence of all-cause death (**Table 1**).

### Characteristics of T2DM Patients and Non-T2DM Patients According to the CysC Level

T2DM and non-T2DM patients were divided into two groups according to the CysC level at a cut-off of 0.93 mg/dL.

Among T2DM patients with CysC >0.93 mg/dL, we noted a higher prevalence of severe pneumonia, heart failure, all-cause death, and older age than those in T2DM patients with CysC ≤0.93 mg/dL. The difference in the number of men, comorbidities (cerebral diseases, cardiovascular diseases, chronic renal diseases, and pulmonary diseases), severe inflammation, diabetes duration, medicine control for diabetes, glucocorticoid use, and DoHS between these two groups was not significant (**Table 2**).

**Table 2:** Characteristics of T2DM patients and non-T2DM patients according to the CysC level.

Variables	Total	CysC ≤ 0.93 mg/dl	CysC ＞ 0.93 mg/dl	p
T2DM	N = 675	N = 344	N = 331	
Age, y	61 (47.5, 68)	56 (41, 65)	63 (55, 71)	<0.001
Men, n (%)	328 (49)	165 (48)	163 (49)	0.798
Severe pneumonia, n (%)	69 (10.2)	14 (4.1)	55 (16.6)	<0.001
Hospital stays (days)	14 (11, 17)	14 (11, 17)	14 (10.5, 18)	0.842
Diabetes duration, y	4 (1, 7)	4 (0.5, 6)	4 (1, 7)	0.305
Medicine control for diabetes				0.310
No medication, n (%)	405 (60)	219 (63.7)	186 (56.2)	
Oral medication, n (%)	205 (30.5)	96 (28.0)	109 (33.0)	
Insulin, n (%)	83 (12.3)	39 (11.4)	41 (13.2)	
Glucocorticoid use				0.271
0 (no)	603 (89.3)	306 (89.0)	297 (89.7)	
1 (yes)	72 (10.7)	38 (11.0)	34 (10.3)	
Comorbidities				
Cerebral diseases, n (%)				0.376
0 (no)	632 (93.6)	324 (94.1)	308 (93.1)	
1 (yes)	43 (6.4)	20 (5.9)	23 (6.9)	
Cardiovascular diseases, n (%)				0.051
0 (no)	388 (57.5)	206 (59.9)	182 (55.0)	
1 (yes)	287 (42.5)	138 (40.1)	149 (45.0)	
Chronic renal diseases, n (%)				0.056
0 (no)	656 (97.2)	565 (98.8)	656 (97.2)	
1 (yes)	19 (2.8)	9 (2.6)	10 (3.0)	
Complications during hospitalization				
Liver injury, n (%)				0.332
0 (no)	503 (75)	260 (76)	243 (73)	
1 (yes)	172 (25)	84 (24)	88 (27)	
Heart failure, n (%)				0.033
0 (no)	655 (97)	339 (99)	316 (95)	
1 (yes)	20 (3)	5 (1)	15 (5)	
Severe inflammatory, n (%)				0.952
0 (no)	541 (80)	275 (80)	266 (81)	
1 (yes)	134 (20)	69 (20)	65 (19)	
All-cause death, n (%)				<0.001
0 (no)	619 (92)	334 (97)	285 (86.1)	
1 (yes)	56 (8.0)	10 (3.0)	46 (13.9)	
**Non-T2DM**	**N = 572**	**N = 290**	**N = 282**	
Age	66 (55, 72)	60 (48, 67)	70 (62, 77)	<0.001
Men, n (%)	270 (47)	120 (41)	150 (53)	
Severe pneumonia, n (%)	49 (8.6)	18 (6.2)	31 (11.0)	0.034
Hospital stays (days)	16 (14, 17)	16 (14, 17)	16 (13, 18)	0.317
Glucocorticoid use				0.298
0 (no)	520 (91.0)	261 (90.0)	259 (91.8)	
1 (yes)	52 (9.0)	29 (10.0)	23 (8.2)	
Comorbidities				
Cerebral diseases, n (%)				0.062
0 (no)	561 (98)	284 (97.9)	277 (93.6)	
1 (yes)	11 (2.0)	6 (2.1)	5 (1.7)	
Cardiovascular diseases, n (%)				0.058
0 (no)	373 (65.2)	195 (67.0)	178 (63.1)	
1 (yes)	199 (34.8)	95 (33.0)	104 (36.9)	
Chronic renal diseases, n (%)				0.268
0 (no)	565 (98.8)	286 (98.6)	279 (98.9)	
1 (yes)	7 (1.2)	4 (1.4)	3 (1.1)	
Complications during hospitalization				
Liver injury, n (%)				0.896
0	486 (85)	246 (85)	240 (86)	
1	86 (15)	44 (15)	42 (14)	
Heart failure, n (%)				0.003
0	548 (96)	285 (98)	263 (93)	
1	24 (4)	5 (2)	19 (7)	
Severe inflammatory, n (%)				0.937
0	516 (90)	258 (89)	248 (88)	
1	56 (10)	32 (11)	34 (12)	
All-cause death, n (%)				1
0	602 (100)	301 (100)	301 (100)	

Data are reported as mean ± SD, median (IQR) or number and percentage. T2DM, type 2 diabetes mellitus; NT-proBNP, N terminal pro B type natriuretic peptide; CysC, cystatin C; BNP, brain natriuretic peptide. Severe inflammation was defined as the highest neutrophil:lymphocyte ratio >6.11 during hospitalization; liver injury was defined as a level of alanine transaminase >40 U/L at any time during hospitalization; and heart failure was defined as clinical symptoms present with the level of NT-proBNP >300 pg/mL during hospitalization.

Among non-T2DM patients with CysC >0.93 mg/dL, we noted a higher prevalence of men, heart failure, and older age than those in T2DM patients with CysC ≤0.93 mg/dL. The difference in the prevalence of comorbidities (cerebral diseases, cardiovascular diseases, chronic renal diseases, and pulmonary diseases), severe pneumonia, glucocorticoid use, DoHS, liver injury, and severe inflammation between these two groups was not significant (**Table 2**).

The difference in routine hematology, indicators of organ function (heart, liver, kidney), coagulation function, and infection indicators between these two groups with T2DM or without T2DM are shown in **Table S2**.

### Characteristics of T2DM Patients and Non-T2DM Patients Grouped by CysC Rangeability

T2DM patients and non-T2DM patients were divided into two groups according to CysC rangeability.

Among T2DM patients with CysC rangeability >0, we noted a higher prevalence of severe pneumonia, severe inflammation, liver injury, and older age than T2DM patients with CysC rangeability ≤0. The difference in the number of men, DoHS, diabetes duration, medicine control for diabetes, glucocorticoid use, comorbidities (cerebral diseases, cardiovascular diseases, chronic renal diseases, and pulmonary diseases), heart failure, and death between these two groups was not significant (**Table 3**).

**Table 3:** Characteristics of T2DM patients and non-T2DM patients grouped by CysC rangeability.

Variables	Total	CysC rangeability ≤ 0	CysC rangeability ＞ 0	p
T2DM	N = 675	N = 407	N = 268	
Age, years	61 (47.5, 68)	56 (41, 65)	63 (55, 71)	<0.001
Men, n (%)	328 (49)	193 (47.4)	135 (50.4)	
Severe pneumonia, n (%)	69 (10.2)	12 (2.9)	57 (21.3)	<0.001
Hospital stays (days)	14 (11, 17)	14 (10, 17)	14 (11, 18)	0.071
Diabetes duration, y	4 (1, 7)	4 (1,6)	5(2, 7)	0.293
Medicine control for diabetes				0.237
No medication, n (%)	405 (60.0)	248 (61.0)	157 (58.6)	
Oral medication, n (%)	205 (30.5)	130 (32.0)	75 (28.0)	
Insulin, n (%)	83 (12.3)	56 (13.7)	27 (10.0)	
Glucocorticoid use				0.112
0 (no)	603 (89.3)	361 (88.6)	242 (90.3)	
1 (yes)	72 (10.7)	46 (11.4)	26 (9.7)	
Comorbidities				
Cerebral diseases, n (%)				0.237
0 (no)	632 (93.6)	380 (93.3)	252 (94.0)	
1 (yes)	43 (6.4)	27 (6.7)	16 (6.0)	
Cardiovascular diseases, n (%)				0.549
0 (no)	388 (57.5)	232 (57.0)	156 (58.3)	
1 (yes)	287 (42.5)	175 (43.0)	112 (41.7)	
Chronic renal diseases, n (%)				0.576
0 (no)	656 (97.2)	395 (97.0)	261 (97.4)	
1 (yes)	19 (2.8)	12 (3.0)	7 (2.6)	
Complications during hospitalization				
Liver injury, n (%)				0.033
0 (no)	503 (75.0)	365 (89.7)	224 (83.6)	
1 (yes)	172 (25.0)	42 (10.3)	44 (16.4)	
Heart failure, n (%)				0.685
0 (no)	655 (97)	396 (97.3)	259 (96.6)	
1 (yes)	20 (3)	11 (2.7)	9 (3.4)	
Severe inflammation, n (%)				0.035
0 (no)	541 (85)	392 (96.3)	249 (92.9)	
1 (yes)	134 (20)	15 (3.7)	19 (7.1)	
All-cause Death, n (%)				<0.001
0 (no)	619 (92)	395 (97.1)	224 (83.6)	
1 (yes)	56 (8)	12 (2.9)	44 (16.4)	
**Non-T2DM**	**N = 572**	**N = 372**	**N = 200**	
Age	66 (55, 72)	60 (48, 67)	70 (62, 77)	<0.001
Men, n (%)	270 (47)	176 (45.6)	105 (48.6)	0.477
Severe pneumonia, n (%)	49 (8.6)	15 (3.9)	34 (17)	<0.001
Hospital stays (days)	16 (14, 17)	16 (9, 17)	16 (14, 18)	0.004
Glucocorticoid use				0.380
0 (no)	520 (91.0)	337 (90.5)	183 (91.5)	
1 (yes)	52 (9.0)	35 (9.5)	17 (8.5)	
Comorbidities				
Cerebral diseases, n (%)				0.479
0 (no)	561 (98)	365 (98.1)	196 (98.0)	
1 (yes)	11 (2.0)	7 (1.9)	4 (2.0)	
Cardiovascular diseases, n (%)				0.067
0 (no)	373 (65.2)	234 (62.8)	133 (66.5)	
1 (yes)	199 (34.8)	138 (37.2)	67 (33.5)	
Chronic renal diseases, n (%)				0.078
0 (no)	565 (98.8)	368 (98.9)	197 (98.5)	
1 (yes)	7 (1.2)	4 (1.1)	3 (1.5)	
Complications during hospitalization				
Liver injury, n (%)				0.387
0 (no)	486 (95)	318 (85.5)	168 (84.0)	
1 (yes)	86 (15)	54 (14.5)	32 (16.0)	
Heart failure, n (%)				0.754
0 (no)	548 (96)	358 (96.3)	190 (95.0)	
1 (yes)	24 (4)	14 (3.7)	10 (5.0)	
Severe inflammation, n (%)				0.325
0 (no)	516 (90)	341 (90.9)	180 (89.0)	
1 (yes)	56 (10)	34 (9.1)	22 (11.0)	
All-cause death, n (%)				1
0 (no)	602 (100)	301 (100)	301 (100)	

Data are reported as mean ± SD, median (IQR) or number and percentage. T2DM, type 2 diabetes mellitus; NT-proBNP, N terminal pro B type natriuretic peptide; CysC, cystatin C; BNP, brain natriuretic peptide. Severe inflammation was defined as the highest neutrophil:lymphocyte ratio >6.11 during hospitalization; liver injury was defined as a level of alanine transaminase >40 U/L at any time during hospitalization; and heart failure was defined as clinical symptoms present with the level of NT-proBNP >300 pg/mL during hospitalization.

Among non-T2DM patients with CysC rangeability >0, we documented a higher prevalence of severe pneumonia, DoHS, and older age than those with CysC rangeability ≤0. The difference in the prevalence of glucocorticoid use, comorbidities (cerebral diseases, cardiovascular diseases, chronic renal diseases, and pulmonary diseases), liver injury, severe inflammation, and heart failure between these two groups was not significant (**Table 3**).

The difference in routine hematology, heart, organ function (liver, kidney), coagulation function, and infection indicators between these two groups of T2DM and non-T2DM patients is shown in **Table S3**.

### Association Between the CysC Level and Organ Dysfunction and All-Cause Death as Classified by T2DM

**Figure 1:**
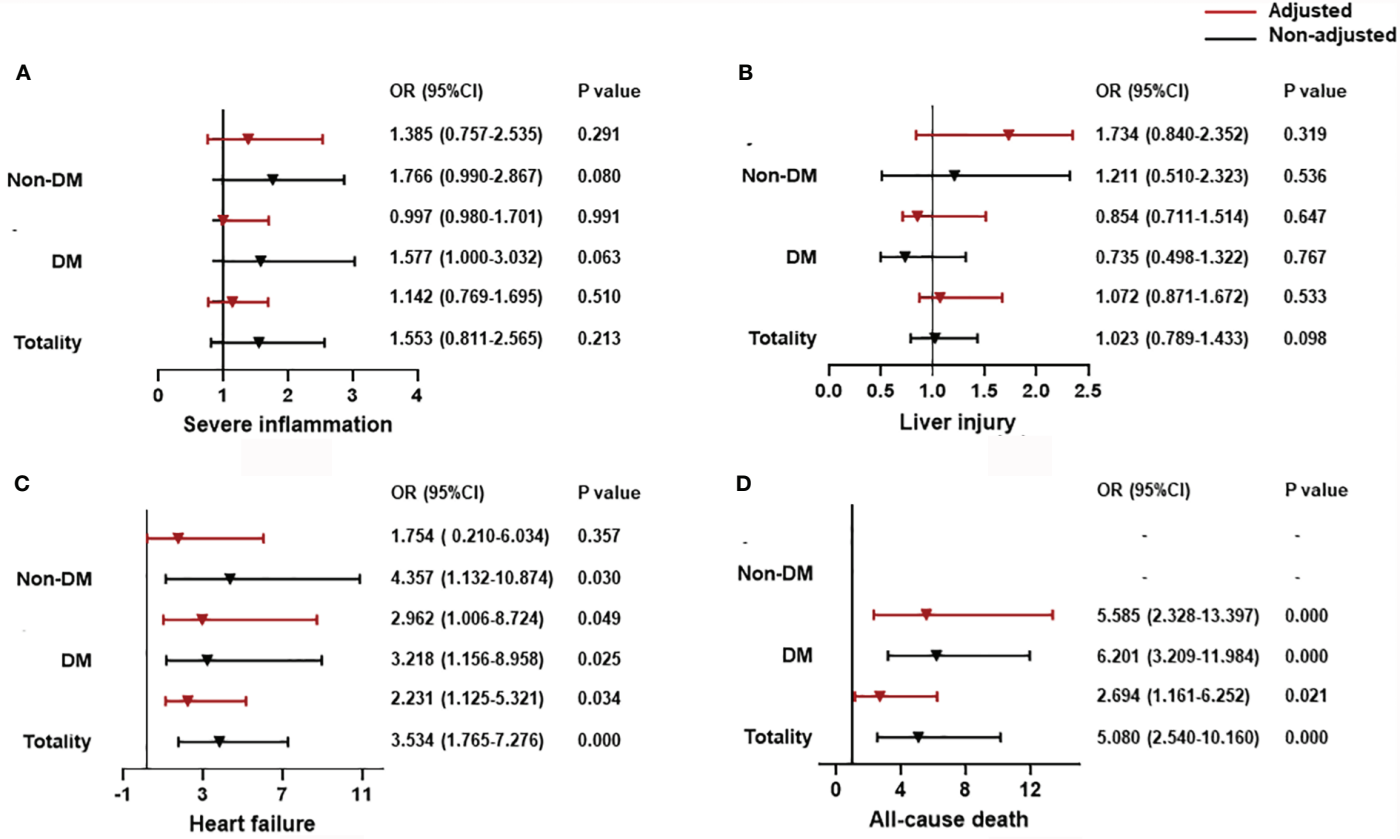
The association between CysC and organ dysfunction and all-cause death classified by T2DM. **(A)** The association between CysC and severe inflammation classified by T2DM. **(B)** The association between CysC and liver injury classified by T2DM. **(C)** The association between CysC and heart failure classified by T2DM. **(D)** The association between CysC and all-cause death classified by T2DM. Severe inflammation was defined as the highest NLR >6.11 during hospitalization; liver injury was defined as a level of ALT >40 U/L at any time during hospitalization; and heart failure was defined as the highest level of NT-proBNP>300 pg/mL during hospitalization. Adjusted by age, severe pneumonia, serum albumin, blood glucose, log NT-proBNP baseline. T2DM, type 2 diabetes mellitus; CysC, cystatin C; NLR, neutrophil-to-lymphocyte ratio; ALT, alanine aminotransferase; NT-proBNP: N terminal pro B type natriuretic peptide.

Multivariate logistic analysis for organ dysfunction and all-cause death showed that if CysC ≤0.93 mg/dL was employed as a cut-off, then CysC >0.93 mg/dL was associated significantly with a risk of heart failure (odds ratio (OR) = 2.231, 95% CI: 1.125–5.321) and all-cause death (2.694, 1.161–6.252), but not with severe inflammation (1.142, 0.769–1.695) or liver injury (1.072, 0.871–1.672) (**Figure 1**).

Using CysC ≤0.93 mg/dL as a cut-off, then CysC >0.93 mg/dL was associated with an increased risk of heart failure (OR = 2.962, 95% CI: 1.006–8.724) and all-cause death (5.585, 2.328–13.397), but not with severe inflammation (0.997, 0.980–1.701) or liver injury (0.854, 0.711–1.514) for T2DM patients. The relationship between CysC >0.93 mg/dL and severe inflammation or liver injury was not significant in the non-T2DM group. CysC >0.93 mg/dL is associated with heart failure (4.357, 1.132-10.874), but adjusted by age, prevalence of severe pneumonia, as well as serum levels of albumin, glucose, and log NT-proBNP, the association between CysC >0.93 mg/dL and heart failure was not significant in the non-T2DM group (**Figure 1**).

**Figure 2:**
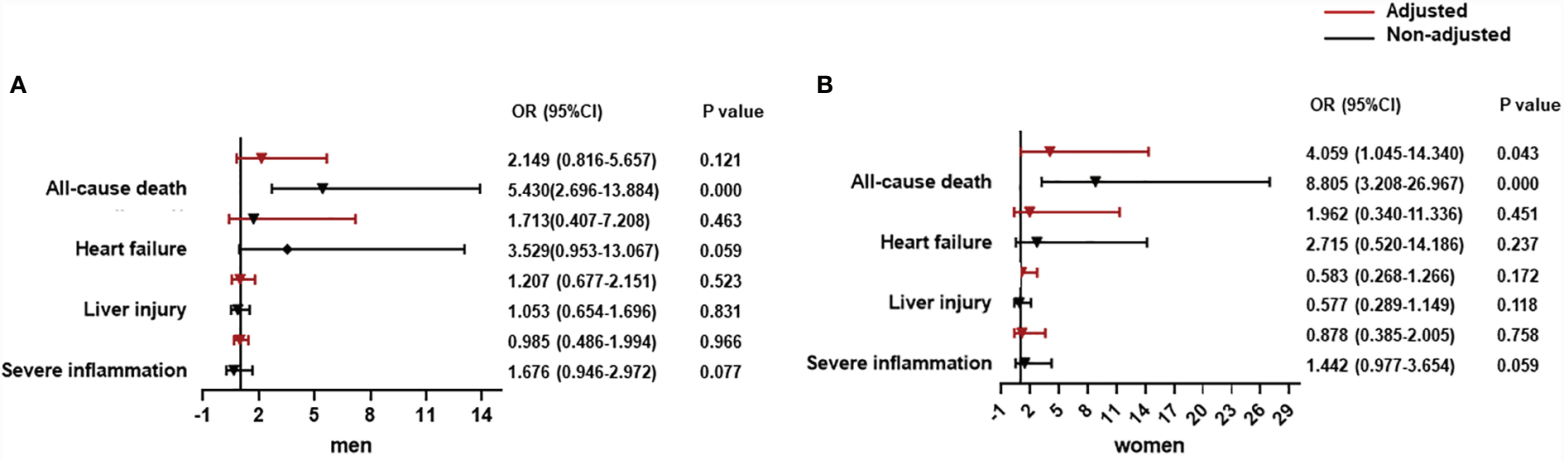
The association between CysC and organ dysfunction and all-cause death in diabetes population classified by sex. **(A)** The association between CysC and organ dysfunction and all-cause death in the male diabetes population. **(B)** The association between CysC and in the female diabetes population. Severe inflammation was defined as the highest NLR >6.11 during hospitalization; liver injury was defined as a level of ALT >40 U/L at any time during hospitalization; and heart failure was defined as the highest level of NT-proBNP>300 pg/mL during hospitalization. Adjusted by age, severe pneumonia, serum albumin, blood glucose, log NT-proBNP baseline. T2DM, type 2 diabetes mellitus; CysC, cystatin C; NLR, neutrophil-to-lymphocyte ratio; ALT, alanine aminotransferase; AST, aspartate aminotransferase; NT-proBNP, N terminal pro B type natriuretic peptide.

In T2DM patients adjusted by age, prevalence of severe pneumonia, as well as serum levels of albumin, glucose, and log NT-proBNP, there was a significantly increased contribution of CysC >0.93 mg/dL to all-cause death (OR = 4.059, 95% CI: 1.045–14.340) in women but not in men. Using a cut-off of CysC ≤0.93 mg/dL, then CysC >0.93 mg/dL did not show an obvious link to severe inflammation, liver injury, or heart failure in men or women (**Figure 2**).

### Association Between CysC Rangeability and All-Cause Death Classified by T2DM

**Figure 3:**
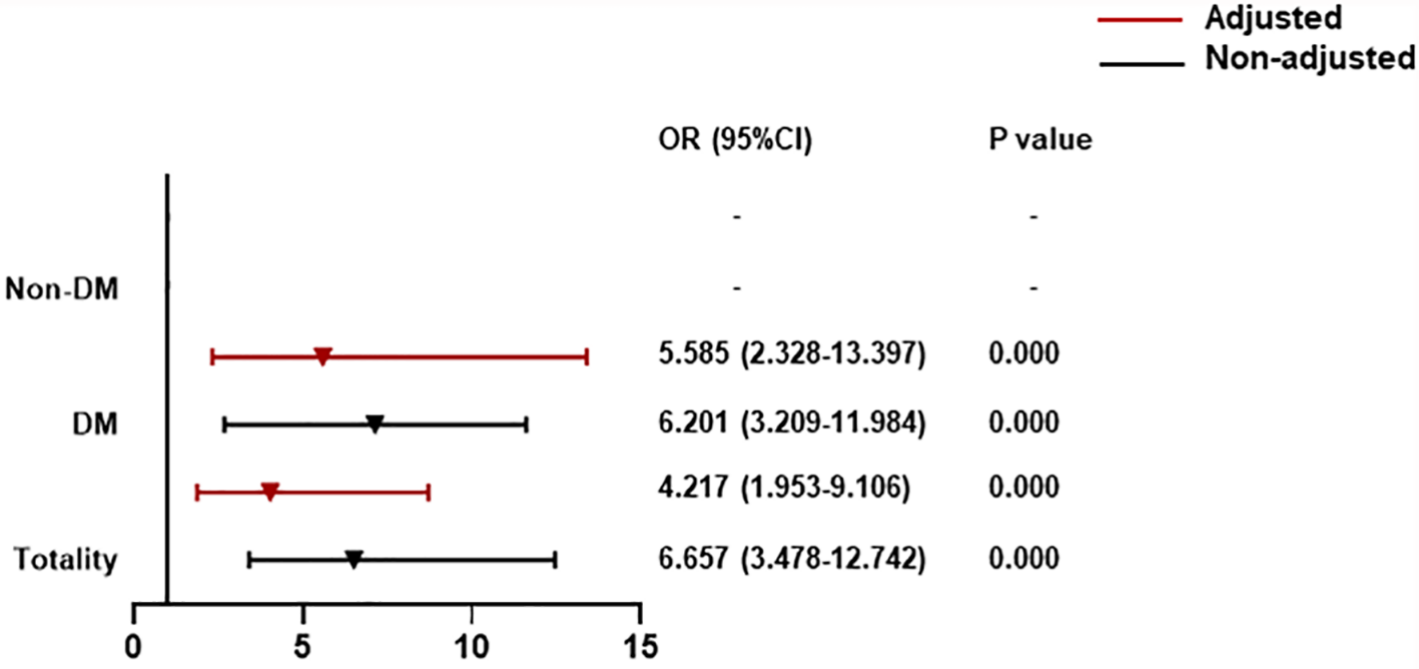
The association between CysC rangeability and all-cause death classified by T2DM. CysC rangeability (changes in renal function) calculated as the difference in the CysC level between the time of hospital admission and the highest CysC level recorded during hospitalization. Adjusted by age, severe pneumonia, serum albumin, blood glucose, log NT-proBNP baseline. T2DM, type 2 diabetes mellitus; NT-proBNP, N terminal pro B type natriuretic peptide.

Compared with patients with CysC rangeability ≤0, those with CysC rangeability >0 had a high correlation with all-cause death (OR = 4.217, 95% CI: 1.953**–**9.106). For T2DM patients, there was a significantly increased contribution of CysC rangeability >0 to all-cause death (OR = 5.585, 95% CI: 2.328**–**13.397) (**Figure 3**).

**Figure 4:**
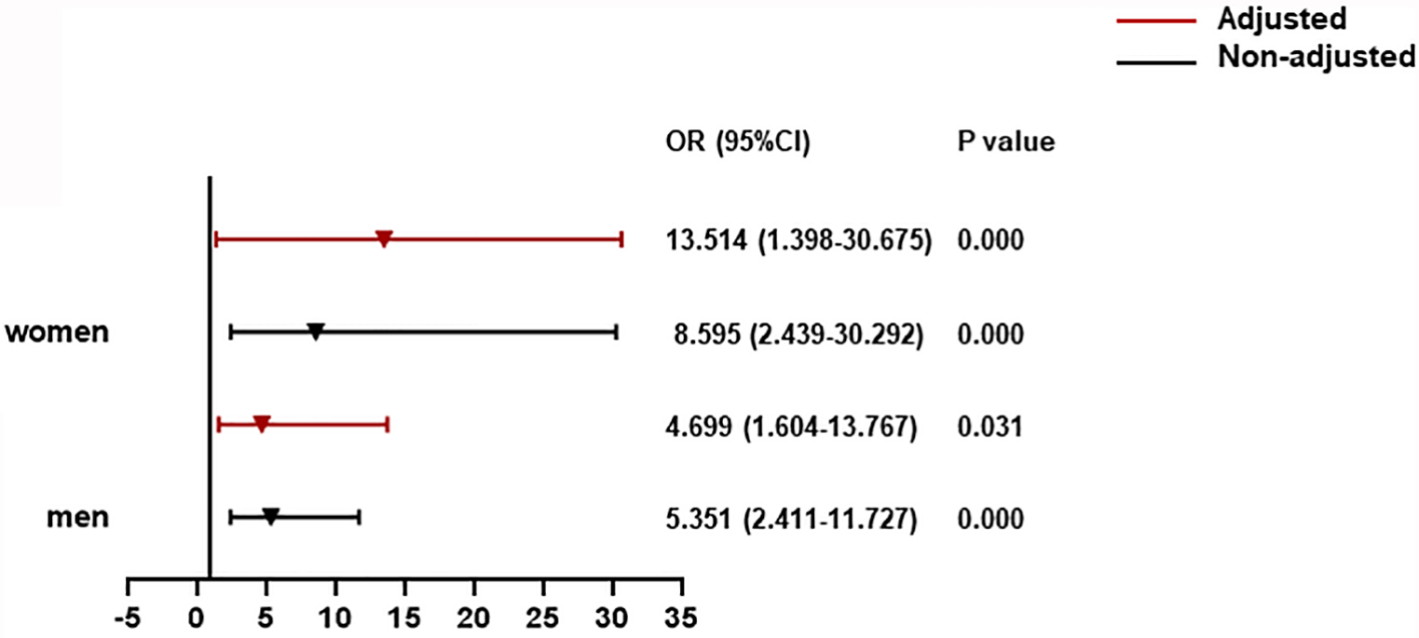
The association between CysC rangeability and all-cause death in T2DM patients. CysC rangeability (changes in renal function) calculated as the difference in the CysC level between the time of hospital admission and the highest CysC level recorded during hospitalization. Adjusted by age, severe pneumonia, serum albumin, blood glucose, log NT-proBNP baseline. T2DM, type 2 diabetes mellitus; NT-proBNP, N terminal pro B type natriuretic peptide.

For T2DM patients adjusted by age, prevalence of severe pneumonia, as well as serum levels of albumin, glucose, and log NT-proBNP, there was a significant contribution of CysC rangeability >0 to all-cause death for men (OR = 4.699, 95% CI: 1.604**–**13.767). Notably, this association was increased significantly for women (OR = 13.514, 95% CI: 1.398**–**30.675) (**Figure 4**).

## Discussion

We found that CysC >0.93 mg/dL and CysC rangeability >0 were independently associated with mortality among COVID-19-associated hospitalized adults with T2DM, and that CysC rangeability contributed a greater risk for in-hospital death than a single concentration of CysC. More importantly, when classified by sex, this association was stronger in women. Notably, we also observed these associations in patients with mild pneumonia, and all causes of death were contributed by T2DM.

No studies have looked at the association between CysC level and all-cause death in COVID-19-associated hospitalized adults, or the effect of a combination of T2DM and sex.

CysC is an endogenous biomarker of renal function. It can be used to indirectly assess the glomerular filtration rate (20), which has been shown to be useful in diagnosing AKI and predicting its outcomes (15). But there remain a few controversial issues, a study by Hamed HM et al. in 2013 included 32 critically ill children who were at risk for developing AKI with a high rate of multi-organ system failure (34.4%) and mortality (62.5%) (21). The study concluded an opposite perspective that CysC is a poor biomarker for diagnosing acute kidney injury. Why the conclusion of this study is different from other studies which included adult patients is uncertain (13, 15, 22), but one reason may be that these children were all critically ill patients with a high incidence of multiple organ dysfunction and death, but the use of glucocorticoids as treatment was not mentioned, however glucocorticoid use can affect serum CysC levels (11). And Hamed HM’s study included neonatal patients whose minimum age is 1 month. Whereas in a review study, Jason H and colleagues concluded that the pathophysiology of neonatal AKI is unique and can be affected by high plasma renin activity, high renal vascular resistance, low baseline GFR, ongoing tubular development, and nephrogenesis (23). Furthermore, compared with older adults with diminishing nephron mass, children’s ongoing growth, greater renal reserve, and superior renal regenerative potential make AKI in children and adults very different (24). Finally, children have unique comorbidities, of which there are no adult equivalents. Comorbidities may affect the course and management of their AKI, such as bronchopulmonary dysplasia, patent ductus arteriosus, and necrotizing enterocolitis (25). Our study included adults with a median age of 63 (interquartile range [51, 70]), and we also analyzed the data of glucocorticoid use and found that glucocorticoid use showed no difference between the T2DM group and non-T2DM group. The difference of glucocorticoid use between low and high CysC groups and low and high CysC-rangeability groups was also no statistically significant. Thus, considering CysC as a superior biomarker of renal function is reasonable in our study.

CysC is produced in nucleated cells, and is influenced less by reduced muscle mass, acute/chronic illness, and nutritional status than creatinine (26). CysC has been found to be a sensitive serum marker of the glomerular filtration rate and a stronger predictor than creatinine as a risk factor of cardiovascular events and all-cause death in older patients (26). Some studies have suggested that a high serum level of CysC is associated with the severity and poor prognosis of cardiovascular disease (27), neurodegeneration (28), chronic kidney disease (29), AKI (30), and chronic liver disease (31) in critically ill ICU patients (32) as well as the general population (33). Thus, CysC could be a molecular marker in serum for predicting a poor prognosis of various acute and chronic diseases.

Since the COVID-19 pandemic, increasing numbers of studies have been devoted to discovering its clinical features and the organ damage it causes. Some studies have shown that organ (heart, liver, kidney) function is impaired in SARS-CoV-2-infected patients (7, 34–36). Moreover, the outcome of COVID-19 patients is associated with organ damage. An observational study from South Korea by Kim YL and colleagues showed that COVID‐19 patients with severe AKI had fatal outcomes (37). Kim YL and colleagues used the rapid development of urea nitrogen and creatinine to evaluate the deterioration of renal function, but not the more stable biomarker CysC. Our study is the first to focus on CysC and CysC rangeability to observe the impact on organ function and mortality in COVID-19 patients.

Researchers have observed that SARS-CoV-2 can directly infect the kidney and cause renal impairment. In an autopsy study, Victor and colleagues quantified the SARS-CoV-2 load in the organs and tissues of 22 patients who had died from COVID-19. They found that the highest number of SARS-CoV-2 copies/cells was detected in the respiratory tract, with lower levels in the kidney, heart, liver, brain, and blood. Those findings indicate that the kidneys are among the most common targets of SARS-CoV-2 (35).

RNA enrichment for transmembrane serine protease 2, angiotensin-converting enzyme 2, and cathepsin L are considered to facilitate SARS-CoV-2 infection. The RNA of genes is enriched in multiple kidney cell types from fetal development through to adulthood. Such enrichment may promote SARS-CoV-2-associated kidney injury (38, 39).

CysC is one of the most reliable parameters of renal function in the general population. Thus, upon SARS-CoV-2 infection, kidney injury can be reflected in the CysC level. Chen X and coworkers studied CTs of the chest in the first week of COVID-19 and CysC levels. They showed that COVID-19 patients with a high CysC level showed more progressive lung lesions on CT. A predictive value of the CysC level upon hospital admission to disease progression was observed rather than dynamic changes (40). Also, COVID-19 severity was closely related to death. We not only found that the CysC level was associated with in-hospital death from COVID-19, we also observed that the change in CysC level during hospitalization was important for predicting death.

Feldman EL and collaborators discovered that T2DM worsened COVID-19 but was also an independent risk factor for severe pneumonia and a poor prognosis (41), data that are in accordance with our results. T2DM can be defined as the phenotype of hyperglycemia that manifests a group of common metabolic disorders. The metabolic disorders related to T2DM cause secondary pathophysiological changes in multiple organs and tissues, and result in various complications that contribute to the morbidity and mortality associated with T2DM (41).

The pathophysiology of SARS-CoV-2 infection is not completely understood. However, studies have demonstrated that SARS-CoV-2 can trigger severe inflammation in certain organs and cause tissue tropism, and shares the same features of chronic inflammation and multi-organ damage observed in T2DM (41). SARS-CoV-2 infection causes intense acute responses to hyperglycemia, inflammation, and tissue damage that are also observed in T2DM. T2DM is characterized by impaired glycemic regulation, chronic low-grade inflammation, slowly progressive multi-organ damage, as well as microvascular (neuropathy, chronic kidney disease) and macrovascular (cerebrovascular disease) complications. Acute COVID-19-associated adverse responses may overlap with glucose instability, preexisting inflammation, and multi-organ damage in T2DM patients and, eventually, worsen outcomes (41). Increased CysC level in COVID-19 patients complicated with AKI is also an indicator for the early detection of diabetic nephropathy in T2DM. Thus, monitoring change in the CysC level may be important for assessing COVID-19 progression.

The CysC level upon hospital admission was strongly associated with death in women, but not in men, in our study. Although the relationship between CysC rangeability and death was not sex-specific, a greater contribution was observed in women than in men. This result may have been because the CysC level is significantly higher in men than that in age-matched women (42), and because if men and women have the same level of CysC, women tend to have worse kidney function. Wang Y and coworkers showed that SARS-CoV-2 infection can aggravate kidney injury in patients with chronic kidney disease (43). Therefore, renal injury in patients with chronic renal disease should be monitored.

Our study had four main limitations. First, it was a single-center observational study, so the generalizability of our results is limited. Second, this study was retrospective. Third, multiple tests for renal function were carried out at different times for each patient. Fourth, biases may have occurred due to the increased number of tests in patients with renal injury. Finally, serum CysC levels are affected by thyroid disease, glucocorticoid use, and obesity (11). However, due to the emergency situation, thyroid diseases were not included in our disease data, and thyroid function of patients in our study were not assessed. Our complication status of T2DM was also deficient.

## Conclusions

CysC may influence the progression and prognosis of COVID-19, and an increased risk of severe complications and death may be seen with COVID-19 patients suffering from T2DM. Attention should be paid to COVID-19 patients with a high level of CysC and CysC rangeability, especially those with T2DM. The exact mechanism underlying CysC-related changes in the course of COVID-19 merit further investigation.

## Data Availability Statement

The datasets used and/or analyzed during the current study are available from the corresponding author on reasonable request.

## Ethics Statement

Written informed consent was obtained from the individual(s), and minor(s)’ legal guardian/next of kin, for the publication of any potentially identifiable images or data included in this article.

## Author Contributions

LY, DX, and YT contributed equally to the study and manuscript. LY, DX, and YT contributed to the conception and design of the study, analysis and interpretation of the data, wrote the manuscript, and approved submission. BL contributed to the data acquisition and analysis. DZ, JW, and HS collected the data. XL and XZ contributed to analysis of the data and provided critical revision of the paper. LZ and ZL provided critical revision of the paper for important intellectual content. All authors contributed to the article and approved the submitted version.

## Funding

This work is partially supported by the National Natural Science Foundation of China under grant no. 61902310, and the Fundamental Research Funds for Central Universities under grant no. xjh012019039.

## Conflict of Interest

Authors HS and XL were employed by company SenseTime Research.

The remaining authors declare that the research was conducted in the absence of any commercial or financial relationships that could be construed as a potential conflict of interest.
