# Supplementary material for: Association Between the Concentration and Rangeability of Cystatin C and Mortality of COVID-19 Patients With or Without Type 2 Diabetes Mellitus: A Retrospective Analysis
Source: Front Endocrinol (Lausanne). 2021 Jun 21;12:642452. doi: 10.3389/fendo.2021.642452 (PMC8256889; doi:10.3389/fendo.2021.642452)
Supplement: Supplementary file 1 [file DataSheet_1.docx]

**Table S1 Laboratory testing index of patients grouped by T2DM**

| Variables | Total  (n = 1247) | Non-T2DM  (n = 572) | T2DM  (n = 675) | p |
| --- | --- | --- | --- | --- |
| CysC baseline, mg/L | 0.93 (0.76, 1.14) | 0.95 (0.79, 1.19) | 0.9 (0.74, 1.12) | < 0.001 |
| Lg NT-proBNP baseline, pg/mL | 1.46 (1.09, 1.84) | 1.55 (1.19, 1.89) | 1.21 (0.38, 1.66) | < 0.001 |
| Lymph_baseline, 10^9^/L | 1.16 (0.82, 1.61) | 1.21 (0.86, 1.62) | 1.1 (0.8, 1.57) | 0.049 |
| Neutrop_baseline, 10^9^/L | 3.24 (2.38, 4.51) | 3.37 (2.50, 4.63) | 3.1 (2.25, 4.41) | 0.001 |
| NLR baseline | 2.73 (1.80, 4.38) | 2.70 (1.82, 4.47) | 3.60 (2.34, 6.05) | < 0.001 |
| PCT_baseline, ug/L | 0.03 (0.03, 0.05) | 0.03 (0.03, 0.04) | 0.03 (0.03, 0.05) | 0.779 |
| ALT_baseline, units/L | 24 (15, 37) | 22 (14, 35) | 26 (16, 39) | 0.003 |
| AST baseline, units/L | 26 (20, 37) | 25 (19, 34) | 27 (21, 39) | 0.037 |
| ALB_baseline, g/L | 37.40 ± 5.59 | 37.52 ± 5.33 | 37.38 ± 5.02 | 0.779 |
| Glu_baseline, mmol/L | 5.43 (4.70, 6.76) | 5.17 (4.61, 6.29) | 5.68 (4.86, 7.2) | < 0.001 |
| Cre_baseline, umol/L | 63.0 (51.3, 79.0) | 61.2 (50.1, 79.8) | 64.7 (52.42, 78.05) | 0.088 |
| ALP_baseline, units/L | 60 (49.0, 74.0) | 62.5 (51.0, 78.0) | 59 (48, 73) | 0.004 |
| CK_baseline, units/L | 68 (45, 120) | 65 (44, 110) | 71 (46, 124.25) | 0.023 |
| CK-MB_baseline, ng/mL | 10 (7, 14) | 10 (7, 13) | 10 (8, 14) | 0.070 |
| cTnI_baseline, ug/L | 0 (0, 0.01) | 0 (0, 0.01) | 0 (0, 0.01) | 0.412 |
| hsCRP_baseline, mg/L | 11.7 (2.1, 20.0) | 6.7 (1.8, 20) | 20 (2.89, 20) | < 0.001 |
| DD_baseline, mg/L | 0.54 (0.24, 1.11) | 0.61 (0.31, 1.69) | 0.57 (0.33, 1.1) | < 0.001 |
| Peak Lymph, 10^9^/L | 1.53 (1.20, 1.92) | 1.49 (1.14, 1.91) | 1.54 (1.25, 1.93) | 0.224 |
| Peak Neutrop, 10^9^/L | 4.74 (3.29, 7.55) | 4.80 (3.40-7.20) | 4.76 (3.19, 7.87) | 0.071 |
| Peak NLR | 1.00 (0.80, 3.75) | 2.16 (1.58, 5.60) | 1.00 (0.87, 2.55) | 0.001 |
| Peak CRP, mg/L | 32.77 (6.80, 99.22) | 15.68 (3.58, 71.67) | 56.58 (10.96, 150.96) | < 0.001 |
| Peak PCT, ug/L | 0.40 (0.12, 2.50) | 0.30 (0.12, 2.46) | 0.48 (0.15, 2.58) | 0.440 |
| Peak ALT, units/L | 46 (27, 82) | 42 (24, 69) | 48 (28, 91) | 0.055 |
| Peak AST, units/L | 35 (24, 64) | 33 (24, 56) | 39 (25,68.75) | 0.067 |
| Peak ALB, g/L | 38.58 ± 5.34 | 38.24 ± 5.94 | 38.69 ± 3.93 | 0.771 |
| Peak Cre, umol/L | 67.3 (54.2, 84.87) | 66.1 (53.43, 80.2) | 69 (55.65, 94.85) | 0.055 |
| Peak ALP, units/L | 74 (60, 93) | 77 (63, 94) | 73 (59, 91) | 0.117 |
| Peak CK, units/L | 71 (49.5, 212.0) | 64 (45, 173) | 85 (53.75, 246.5) | 0.108 |
| Peak CK-MB, ng/mL | 15 (10, 24) | 14 (10, 21) | 14 (10, 26.25) | 0.377 |
| Peak cTnl, ug/L | 0.08 (0.02, 0.48) | 0.06 (0.03, 0.60) | 0.09 (0.02, 0.42) | 0.587 |
| Peak CysC, mg/L | 1.23 (0.98, 1.54) | 1.26 (0.95, 1.59) | 1.17 (0.99, 1.51) | 0.831 |
| Peak hs-CRP, mg/L | 7.70 (2.30, 20.0) | 6.30 (2.10, 20) | 20 (2.35, 27) | 0.263 |
| Peak DD, mg/L | 3.22 (1.02, 6.09) | 2.99 (0.94, 6.34) | 3.43 (0.98, 5.88) | 0.718 |

Data are reported as mean ± SD, median (IQR) or number and percentage. T2DM, Type 2 diabetes mellitus; NT-proBNP: N terminal pro B type natriuretic peptide; NLR, Neutrophil-to-Lymphocyte Ratio; PCT, procalcitonin; ALT, Alanine aminotransferase; AST, Aspartate aminotransferase; ALB, albumin; Glu, glucose; Cre, creatinine; ALP, alkaline phosphatase; CK, choline kinase; CK-MB, creatine kinase isoenzymes; cTnI, hypersensitive troponin I; hsCRP, high-sensitivity C-reactive protein; DD, D-Dimer; Lymph, lymphocyte; Neutrop, Neutrophil; CRP, C-reactive protein; PCT, procalcitonin; ALT, alanine transaminase; CysC, Cystatin C.

**Table S2 Laboratory testing index of patients with or without DM classified by cystatin-C level**

| Variables | Total | CysC low | CysC high | p |
| --- | --- | --- | --- | --- |
| **T2DM** | **N = 675** | **N = 344** | **N = 331** |  |
| CysC baseline, mg/L | 0.9 (0.74, 1.12) | 0.84 (0.71, 1) | 0.99 (0.78, 1.18) | < 0.001 |
| Lg NT-proBNP baseline, pg/mL | 1.21 (0.38, 1.66) | 1.09 (0, 1.57) | 1.32 (0.65, 1.75) | < 0.001 |
| Lymph baseline, 10^9^/L | 1.1 (0.8, 1.57) | 1.16 (0.8, 1.63) | 1.05 (0.79, 1.52) | 0.057 |
| Neutrop baseline, 10^9^/L | 3.1 (2.25, 4.41) | 3.05 (2.24, 4.32) | 3.14 (2.33, 4.62) | 0.219 |
| NLR baseline | 2.75 (1.77, 4.27) | 2.49 (1.66, 3.63) | 3.11 (1.93, 5.39) | 0.002 |
| PCT baseline, ug/L | 0.03 (0.03, 0.05) | 0.03 (0.03, 0.03) | 0.03 (0.03, 0.05) | 0.048 |
| ALT baseline, units/L | 26 (16, 39) | 26 (17, 37) | 26 (16, 39) | 0.838 |
| AST baseline, units/L | 27 (21, 39) | 27 (20, 39) | 28 (21, 38) | 0.566 |
| ALB baseline, g/L | 37.38 ± 5.02 | 38.25 ± 4.89 | 36.48 ± 5.01 | < 0.001 |
| Glu baseline, mmol/L | 5.68 (4.86, 7.2) | 5.51 (4.74, 7.19) | 5.87 (4.98, 7.23) | 0.034 |
| Cre baseline, umol/L | 64.7 (52.42, 78.05) | 63.5 (52.15, 76.07) | 65.85 (53.48, 81.62) | 0.146 |
| ALP baseline, units/L | 59 (48, 73) | 58 (47.25, 72) | 59 (49, 73) | 0.165 |
| CK baseline, units/L | 71 (46, 124.25) | 70 (47, 119) | 72 (46, 130.5) | 0.716 |
| CK-MB_baseline, ng/mL | 10 (8, 14) | 10 (7, 14) | 11 (8, 14) | 0.069 |
| cTnI baseline, ug/L | 0 (0, 0.01) | 0 (0, 0.01) | 0 (0, 0.01) | < 0.001 |
| hsCRP baseline, mg/L | 20 (2.89, 20) | 20 (1.9, 20) | 20 (4.03, 20) | 0.015 |
| DD baseline, mg/L | 0.57 (0.33, 1.1) | 0.51 (0.29, 0.84) | 0.66 (0.37, 1.49) | < 0.001 |
| Peak Lymph, 10^9^/L | 1.54 (1.25, 1.93) | 1.52 (1.27, 1.88) | 1.55 (1.21, 1.94) | 0.988 |
| Peak Neutrop, 10^9^/L | 4.76 (3.19, 7.88) | 5.03 (3.14, 7.72) | 4.59 (3.21, 8.51) | 0.216 |
| Peak NLR | 1.00 (0.87, 2.51) | 1.00 (1.00, 2.31) | 1.00 (0.57, 2.74) | 0.985 |
| Peak CRP, mg/L | 56.58 (10.96, 150.96) | 52.42 (7.98, 156.88) | 65.06 (13.83, 138.53) | 0.336 |
| Peak PCT, ug/L | 0.48 (0.15, 2.58) | 0.54 (0.18, 2.72) | 0.36 (0.12, 2.58) | 0.372 |
| Peak ALT, units/L | 48 (28, 91) | 48 (31, 107) | 48 (25, 84.5) | 0.204 |
| Peak AST, units/L | 39 (25, 68.75) | 37 (25, 70) | 40 (25.75, 66) | 0.568 |
| Peak ALB, g/L | 38.69 ± 3.93 | 39.42 ± 3.91 | 38.09 ± 3.85 | 0.011 |
| Peak Cre, umol/L | 69 (55.65, 94.85) | 66.8 (55, 89.38) | 72.1 (56, 98.2) | 0.428 |
| Peak ALP, units/L | 73 (59, 91) | 70 (55.25, 95.25) | 74 (61.5, 89.5) | 0.301 |
| Peak CK, units/L | 85 (53.75, 246.5) | 73 (51, 244) | 108 (54, 263.5) | 0.917 |
| Peak CK-MB, ng/mL | 14 (10, 26.25) | 14.5 (9, 27.25) | 13.5 (11, 24.5) | 0.989 |
| Peak cTnl, ug/L | 0.09 (0.02, 0.42) | 0.1 (0.01, 0.42) | 0.08 (0.02, 0.48) | 0.86 |
| Peak CysC, mg/L | 1.17 (0.99, 1.51) | 1.12 (0.97, 1.4) | 1.21 (1.02, 1.64) | 0.052 |
| Peak hs-CRP, mg/L | 20 (2.35, 27) | 8.02 (1.52, 27.85) | 20 (4.19, 20) | 0.346 |
| Peak DD, mg/L | 3.43 (0.98, 5.88) | 3.96 (0.98, 7.24) | 2.88 (1.01, 5.64) | 0.292 |
| **Non-T2DM** | **N = 572** | **N = 290** | **N = 282** |  |
| CysC baseline, mg/L | 0.95 (0.79, 1.19) | 0.78 (0.70, 0.85) | 1.17 (1.01, 1.54) | < 0.001 |
| Lg NT-proBNP baseline, pg/mL | 1.55 (1.19, 1.89) | 1.43 (1.07, 1.70) | 1.64 (1.34, 2.05) | < 0.001 |
| Lymph baseline, 10^9^/L | 1.21 (0.86, 1.62) | 1.30 (0.93, 1.62) | 1.15 (0.82, 1.63) | 0.007 |
| Neutrop baseline, 10^9^/L | 3.37 (2.50, 4.63) | 3.18 (2.37, 4.32) | 3.58 (2.67, 4.88) | 0.254 |
| NLR baseline | 2.70 (1.82, 4.47) | 2.51 (1.76, 3.72) | 2.97 (1.88, 5.09) | 0.212 |
| PCT baseline, ug/L | 0.03 (0.03, 0.04) | 0.03 (0.03, 0.03) | 0.03 (0.03, 0.09) | 0.203 |
| ALT baseline, units/L | 22 (14, 35) | 24 (15, 37) | 21 (14, 33) | 0.002 |
| AST baseline, units/L | 25 (19, 34) | 26 (20, 34) | 24 (18, 34) | < 0.001 |
| ALB baseline, g/L | 37.52 ± 5.33 | 37.99 ± 5.40 | 37.24 ± 5.18 | < 0.001 |
| Glu_baseline, mmol/L | 5.17 (4.61, 6.29) | 5.24 (4.61, 6.41) | 5.12 (4.62, 6.20) | 0.144 |
| Cre baseline, umol/L | 61.2 (50.1, 79.8) | 53.4 (45.1, 63.2) | 73.7 (58.1, 95.3) | < 0.001 |
| ALP baseline, units/L | 62.5 (51.0, 78.0) | 57.0 (48.0, 70.0) | 66 (54, 82) | 0.132 |
| CK baseline, units/L | 65 (44, 110) | 66 (45, 104) | 65 (44, 113) | 0.030 |
| CK-MB_baseline, ng/mL | 10 (7, 13) | 9 (7, 12) | 10 (7, 14) | 0.014 |
| cTnI baseline, ug/L | 0 (0, 0.01) | 0 (0, 0.01) | 0.01 (0, 0.01) | < 0.001 |
| hsCRP baseline, mg/L | 6.7 (1.8, 20) | 4.9 (1.51, 20) | 8.12 (2.11, 20) | < 0.001 |
| DD baseline, mg/L | 0.61 (0.31, 1.69) | 0.6 (0.35, 1.18) | 0.82 (0.38, 3.10) | < 0.001 |
| Peak Lymph, 10^9^/L | 1.49 (1.14, 1.91) | 1.55 (1.16, 2.03) | 1.59 (1.08, 1.99) | 0.377 |
| Peak Neutrop, 10^9^/L | 4.80 (3.40-7.20) | 4.68 (3.26, 6.45) | 4.78 (3.66, 8.12) | 0.800 |
| Peak NLR | 2.16 (1.58, 5.60) | 1.88 (0.45, 7.60) | 2.45 (1.16, 5.34) | 0.417 |
| Peak CRP, mg/L | 15.68 (3.58, 71.67) | 9.65 (4.00, 41.32) | 26.14 (6.07, 79.44) | 0.065 |
| Peak PCT, ug/L | 0.30 (0.12, 2.46) | 0.38 (0.15, 2.67) | 0.2 (0.1, 1.18) | 0.387 |
| Peak ALT, units/L | 42 (24, 69) | 36 (23, 68) | 48 (27.9, 80) | 0.098 |
| Peak AST, units/L | 33 (24, 56) | 29.0 (21.0, 53.0) | 38.0 (28.0, 68.0) | 0.003 |
| Peak ALB, g/L | 38.24 ± 5.94 | 38.90 ± 5.28 | 38.76 ± 5.42 | 0.187 |
| Peak Cre, umol/L | 66.1 (53.43, 80.2) | 65.0 (52.0, 73.0) | 70.1 (55.2, 84.7) | 0.009 |
| Peak ALP, units/L | 77 (63, 94) | 76.5 (65.0, 90.0) | 76 (61, 99) | 0.608 |
| Peak CK, units/L | 64 (45, 173) | 53 (45.0, 76.0) | 75 (47, 218) | 0.211 |
| Peak CK-MB, ng/mL | 14 (10, 21) | 11 (9, 20) | 14 (11, 22) | 0.030 |
| Peak cTnl, ug/L | 0.06 (0.03, 0.60) | 0.02 (0.01, 0.14) | 0.09 (0.03, 0.93) | 0.113 |
| Peak CysC, mg/L | 1.26 (0.95, 1.59) | 1.11 (0.91, 1.36) | 1.3 (1.10, 1.88) | 0.03 |
| Peak hs-CRP, mg/L | 6.30 (2.10, 20) | 2.56 (1.66, 6.34) | 20 (4.53, 20) | < 0.001 |
| Peak DD, mg/L | 2.99 (0.94, 6.34) | 1.68 (0.89, 5.09) | 4.7 (1.30, 7.20) | 0.001 |

Data are reported as mean ± SD, median (IQR) or number and percentage. T2DM, Type 2 diabetes mellitus; NT-proBNP: N terminal pro B type natriuretic peptide; NLR, Neutrophil-to-Lymphocyte Ratio; PCT, procalcitonin; ALT, Alanine aminotransferase; AST, Aspartate aminotransferase; ALB, albumin; Glu, glucose; Cre, creatinine; ALP, alkaline phosphatase; CK, choline kinase; CK-MB, creatine kinase isoenzymes; cTnI, hypersensitive troponin I; hsCRP, high-sensitivity C-reactive protein; DD, D-Dimer; Lymph, lymphocyte; Neutrop, Neutrophil; CRP, C-reactive protein; PCT, procalcitonin; ALT, alanine transaminase; CysC, Cystatin C.

**Table S3 Laboratory testing index of patients with or without T2DM classified by CysC rangeability**

| Variables | Total | CysC rangeability ≤0 | CysC rangeability＞0 | p |
| --- | --- | --- | --- | --- |
| **T2DM** | **N = 675** | **N = 407** | **N = 268** |  |
| CysC baseline, mg/L | 0.96 (0.79, 1.19) | 0.9 (0.76, 1.06) | 1.02 (0.84, 1.28) | < 0.001 |
| Lg NT-proBNP baseline, pg/mL | 1.4 (0.77, 1.8) | 1.23 (0.08, 1.68) | 1.5 (0.98, 1.94) | < 0.001 |
| Lymph_baseline, 10^9^/L | 1.19 (0.86, 1.62) | 1.28 (0.88, 1.68) | 1.11 (0.82, 1.54) | 0.008 |
| Neutrop_baseline, 10^9^/L | 3.37 (2.54, 4.64) | 3.29 (2.5, 4.49) | 3.46 (2.57, 4.81) | 0.287 |
| NLR baseline | 2.50 (1.70, 3.68) | 2.24 (1.62, 3.09) | 3.34 (2.42, 5.93) | 0.206 |
| PCT baseline, ug/L | 0.25 (0.25, 0.25) | 0.25 (0.25, 0.25) | 0.25 (0.25, 0.58) | 0.163 |
| ALT baseline, units/L | 26 (16, 39) | 24 (16, 38) | 26 (18, 40) | 0.002 |
| AST baseline, units/L | 27 (21, 38) | 26 (20, 37) | 30 (21, 42) | 0.000 |
| ALB baseline, g/L | 37.15 ± 4.33 | 37.64 ± 4.21 | 36.25 ± 4.76 | < 0.001 |
| Glu baseline, mmol/L | 5.67 (4.84, 7.19) | 5.62 (4.87, 7.13) | 5.76 (4.80, 7.33) | 0.135 |
| Cre baseline, umol/L | 65.10 (52.40, 78.70) | 64.20 (51.60, 78.20) | 65.5 (54.40, 80.80) | < 0.001 |
| ALP baseline, units/L | 59.00 (48.00, 73.00) | 57.00 (47.00, 72.00) | 61.00 (49.00, 74.00) | 0.101 |
| CK baseline, units/L | 72.00 (46.00, 129.00) | 68.00 (45.00, 123.00) | 76.50 (48.25, 136.75) | 0.023 |
| CK-MB_baseline, ng/mL | 10 (8, 14) | 10 (8, 13) | 11 (7, 14) | 0.01 |
| cTnI baseline, ug/L | 0 (0, 0.01) | 0 (0, 0.01) | 0.01 (0, 0.01) | < 0.001 |
| hsCRP baseline, mg/L | 20.0 (2.8, 20.0) | 20.0 (2.30, 20.0) | 20.0 (3.14, 20.0) | < 0.001 |
| DD baseline, mg/L | 0.69 (0.39, 2.15) | 0.6 (0.35, 1.18) | 0.92 (0.45, 3.12) | < 0.001 |
| Peak Lymph, 10^9^/L | 1.53 (1.25, 1.93) | 1.54 (1.23, 1.93) | 1.53 (1.25, 1.93) | 0.245 |
| Peak Neutrop, 10^9^/L | 4.76 (3.19, 7.88) | 4.81 (3.16, 7.63) | 4.66 (3.42, 9.38) | 0.803 |
| Peak NLR | 1.00 (0.87, 2.51) | 1.00 (1.00, 2.40) | 1.00 (1.00, 2.92) | 0.611 |
| Peak CRP, mg/L | 56.58 (10.38, 152.32) | 58.76 (12.75, 149.93) | 52.43 (8.65, 138.53) | 0.027 |
| Peak PCT, ug/L | 0.48 (0.15, 2.69) | 0.37 (0.10, 4.34) | 0.51 (0.18, 2.43) | 0.901 |
| Peak ALT, units/L | 49.5 (28.25, 96.75) | 43.00 (28.00, 84.50) | 54.00 (29.00, 107.00) | 0.058 |
| Peak AST, units/L | 39 (25, 68.75) | 35 (24, 59) | 44 (27, 77.5) | 0.066 |
| Peak ALB, g/L | 38.69 ± 3.93 | 38.60 ± 3.79 | 38.25 ± 4.04 | 0.691 |
| Peak Cre, umol/L | 69 (55.65, 94.85) | 67.8 (54.1, 100.1) | 74.5 (59.6, 105.0) | 0.280 |
| Peak ALP, units/L | 73 (59, 91) | 70.50 (59.00, 85.25) | 75.00 (59.00, 101.50) | 0.021 |
| Peak CK, units/L | 85 (53.75, 246.5) | 85 (45.00, 398.00) | 115 (63, 459) | 0.522 |
| Peak CK-MB, ng/mL | 14.0 (10, 26.25) | 14.0 (9, 27.25) | 15.0 (10.0, 31.0) | 0.338 |
| Peak cTnl, ug/L | 0.09 (0.02, 0.42) | 0.1 (0.98, 1.52) | 1.20 (1.00, 1.50) | 0.909 |
| Peak CysC, mg/L | 1.17 (0.99, 1.51) | 1.15 (0.97, 1.4) | 1.21 (1.02, 1.64) | 0.717 |
| Peak hs-CRP, mg/L | 20 (2.35, 27) | 8.02 (1.52, 27.85) | 20 (4.19, 20) | 0.297 |
| Peak DD, mg/L | 3.43 (0.98, 5.88) | 3.11 (0.97, 5.48) | 3.70 (1.04, 7.21) | 0.936 |
| **Non-T2DM** | **N = 572** | **N = 372** | **N = 200** |  |
| CysC baseline, mg/L | 0.95 (0.79, 1.19) | 0.98 (0.82, 1.20) | 0.88 (0.74, 1.16) | < 0.001 |
| LgBNP baseline, pg/mL | 1.55 (1.19, 1.89) | 1.53 (1.17, 1.85) | 1.58 (1.28, 1.99) | < 0.001 |
| Lymph baseline, 10^9^/L | 1.21 (0.86, 1.62) | 1.34 (1.01, 1.72) | 0.94 (0.68, 1.28) | 0.006 |
| Neutrop baseline, 10^9^/L | 3.37 (2.50, 4.63) | 3.29 (2.5, 4.37) | 3.68 (2.58, 5.40) | 0.334 |
| NLR baseline | 2.70 (1.82, 4.47) | 2.35 (1.70, 3.57) | 3.70 (2.54, 6.10) | 0.03 |
| PCT baseline, ug/L | 0.03 (0.03, 0.04) | 0.25 (0.25, 0.25) | 0.25 (0.25, 0.90) | 0.322 |
| ALT baseline, units/L | 22 (14, 35) | 20 (13, 32) | 27 (18, 44) | 0.003 |
| AST baseline, units/L | 25 (19, 34) | 23 (18, 31) | 30 (22, 47) | < 0.001 |
| ALB baseline, g/L | 37.52 ± 5.33 | 38.60 ± 5.24 | 35.57 ± 4.96 | < 0.001 |
| Glu baseline, mmol/L | 5.17 (4.61, 6.29) | 5.06 (4.54, 6.12) | 5.37 (4.70, 6.59) | 0.040 |
| Cre baseline, umol/L | 61.2 (50.1, 79.8) | 59.80 (49.00, 76.40) | 65.5 (51.6, 86.8) | < 0.001 |
| ALP baseline, units/L | 62.5 (51.0, 78.0) | 60.00 (49.00, 75.00) | 67.0 (54.0, 81.0) | 0.099 |
| CK baseline, units/L | 65 (44, 110) | 60.00 (44.00, 98.00) | 79.0 (49.0, 152.0) | 0.031 |
| CK-MB_baseline, ng/mL | 10 (7, 13) | 9 (7, 12) | 11 (8, 15) | 0.023 |
| cTnI baseline, ug/L | 0 (0, 0.01) | 0 (0, 0.01) | 0.01 (0, 0.01) | < 0.001 |
| hsCRP baseline, mg/L | 6.7 (1.8, 20) | 3.40 (1.30, 20.0) | 20.0 (7.02, 20.0) | < 0.001 |
| DD baseline, mg/L | 0.61 (0.31, 1.69) | 0.55 (0.26, 1.14) | 0.85 (0.41, 3.16) | < 0.001 |
| Peak Lymph, 10^9^/L | 1.49 (1.14, 1.91) | 1.51 (1.18, 1.93) | 1.60 (1.10, 2.03) | 0.334 |
| Peak Neutrop, 10^9^/L | 4.80 (3.40-7.20) | 4.68 (3.61, 6.48) | 4.77 (3.76, 7.68) | 0.753 |
| Peak NLR | 2.16 (1.58, 5.60) | 1.79 (1.20, 5.22) | 2.59 (0.56, 9.05) | 0.067 |
| Peak CRP, mg/L | 15.68 (3.58, 71.67) | 9.67 (2.59, 63.00) | 26.02 (5.37, 70.67) | 0.231 |
| Peak PCT, ug/L | 0.30 (0.12, 2.46) | 0.29 (0.11, 0.78) | 0.78 (0.23, 4.30) | 0.556 |
| Peak ALT, units/L | 42 (24, 69) | 40 (22, 67) | 50 (30, 77) | 0.023 |
| Peak AST, units/L | 33 (24, 56) | 33.0 (22.0, 51.0) | 35.0 (28.0, 64.0) | 0.341 |
| Peak ALB, g/L | 38.24 ± 5.94 | 38.55 ± 4.02 | 38.63 ± 4.22 | 0.886 |
| Peak Cre, umol/L | 66.1 (53.43, 80.2) | 67.07 (55.32, 86.21) | 65.12 (54.1, 81.2) | 0.702 |
| Peak ALP, units/L | 77 (63, 94) | 78.0 (63.0, 91.0) | 75.0 (64.0, 91.0) | 0.832 |
| Peak CK, units/L | 64 (45, 173) | 70.0 (49.0, 245.0) | 71.0 (42.0, 315.0) | 0.812 |
| Peak CK-MB, ng/mL | 14 (10, 21) | 13 (10, 22) | 14 (10, 22) | 0.631 |
| Peak cTnl, ug/L | 0.06 (0.03, 0.60) | 0.04 (0.01, 0.09) | 0.12 (0.05, 1.90) | 0.221 |
| Peak CysC, mg/L | 1.26 (0.95, 1.59) | 1.26 (0.98, 1.60) | 1.23 (1.00, 1.50) | 0.772 |
| Peak hs-CRP, mg/L | 6.30 (2.10, 20) | 6.77 (1.98, 20.00) | 5.76 (2.32, 20.00) | 0.811 |
| Peak DD, mg/L | 2.99 (0.94, 6.34) | 3.72 (1.12, 6.23) | 2.42 (0.81, 6.34) | 0.127 |

Data are reported as mean ± SD, median (IQR) or number and percentage. T2DM, Type 2 diabetes mellitus; NT-proBNP: N terminal pro B type natriuretic peptide; NLR, Neutrophil-to-Lymphocyte Ratio; PCT, procalcitonin; ALT, Alanine aminotransferase; AST, Aspartate aminotransferase; ALB, albumin; Glu, glucose; Cre, creatinine; ALP, alkaline phosphatase; CK, choline kinase; CK-MB, creatine kinase isoenzymes; cTnI, hypersensitive troponin I; hsCRP, high-sensitivity C-reactive protein; DD, D-Dimer; Lymph, lymphocyte; Neutrop, Neutrophil; CRP, C-reactive protein; PCT, procalcitonin; ALT, alanine transaminase; CysC, Cystatin C.
